# Franklin Was a Fool

**Published:** 1872-03

**Authors:** 


### "FRANKLIN WAS A FOOL,"

Said the great statesman, Thomas Jefferson, of the greater philosopher, because the author of "Poor Richard's Almanac," during his last illness, would sit at the open window, from his great confidence in the curative virtues of pure outdoor air. Benjamin Franklin is not the only man whose hobby rode him into the grave.

Morrison had such great faith in his famous pills that he fully believed nothing could save him if they did not; in that faith he took two hundred and fifty-six in one day, and — died before its close.

Alcott and Graham died before their time for want of a good dinner of roast beef or boiled mutton; and how many are watered to death at the various "cures" and "homes" of the "sloshing schools," may never be known. If Franklin had been tucked in, on a good bed, and drank a quart of some old woman's hot catnip tea, until his whole body had steamed in perspiration, instead of having the cold air to close the pores of the skin, and cover him with goose flesh at the very moment he was almost stifling with asthma, he would most likely have lived for many a sunny day afterwards — considering his exemplary temperate habits, might have lived a decade longer, and opened his eyes on the nineteenth century, instead of dying at eighty-four. It is certainly of incalculable advantage for an invalid to breathe a pure air. That alone would cure half of our ordinary ailments. Franklin was right in his appreciation of cool, pure air; his mistake was, and it was fatal to him, that the whole body, the skin, needed warmth; the lungs only required the cool, out-door air. Therefore in nursing the sick, let the air of the room be cool and pure, but let the body of the patient be kept warm in bed. The best way to ventilate a room in summer and keep it cool, is to have a light fire, or a lamp or candle, on the hearth of an open fire-place; in winter let there be a good fire night and day, with the inner door open; in both cases the more impure air of the room is near the floor, and is driven to the fireplace and up the chimney, by the cooler air coming in at the door, — the heat in the fire-place creating a draft up the chimney. The very best, most perfect, and least troublesome arrangement for a thing of this kind is Dixon's device, of Philadelphia, the imitation of a log fire, supplied with flame by a gas pipe, which for cities, and where gas is always at hand, is admirable; or his low-down grate, for summer, and his low-down grate, with a large fire-place, for the winter.

